# Recent Application of Core-Shell Nanostructured Catalysts for CO_2_ Thermocatalytic Conversion Processes

**DOI:** 10.3390/nano12213877

**Published:** 2022-11-02

**Authors:** Nisa Afiqah Rusdan, Sharifah Najiha Timmiati, Wan Nor Roslam Wan Isahak, Zahira Yaakob, Kean Long Lim, Dalilah Khaidar

**Affiliations:** 1 Fuel Cell Institute, Universiti Kebangsaan Malaysia, Bangi 43600, Selangor, Malaysia; 2 Department of Chemical and Process Engineering, Faculty of Engineering and Built Environment, Univesiti Kebangsaan Malaysia, Bangi 43600, Selangor, Malaysia

**Keywords:** core-shell nanostructures, CO_2_ hydrogenation, dry reforming methane, thermocatalytic reactions, sintering, coke formation, methanol synthesis, methanation, mesoporous materials, hydrogen

## Abstract

Carbon-intensive industries must deem carbon capture, utilization, and storage initiatives to mitigate rising CO_2_ concentration by 2050. A 45% national reduction in CO_2_ emissions has been projected by government to realize net zero carbon in 2030. CO_2_ utilization is the prominent solution to curb not only CO_2_ but other greenhouse gases, such as methane, on a large scale. For decades, thermocatalytic CO_2_ conversions into clean fuels and specialty chemicals through catalytic CO_2_ hydrogenation and CO_2_ reforming using green hydrogen and pure methane sources have been under scrutiny. However, these processes are still immature for industrial applications because of their thermodynamic and kinetic limitations caused by rapid catalyst deactivation due to fouling, sintering, and poisoning under harsh conditions. Therefore, a key research focus on thermocatalytic CO_2_ conversion is to develop high-performance and selective catalysts even at low temperatures while suppressing side reactions. Conventional catalysts suffer from a lack of precise structural control, which is detrimental toward selectivity, activity, and stability. Core-shell is a recently emerged nanomaterial that offers confinement effect to preserve multiple functionalities from sintering in CO_2_ conversions. Substantial progress has been achieved to implement core-shell in direct or indirect thermocatalytic CO_2_ reactions, such as methanation, methanol synthesis, Fischer–Tropsch synthesis, and dry reforming methane. However, cost-effective and simple synthesis methods and feasible mechanisms on core-shell catalysts remain to be developed. This review provides insights into recent works on core-shell catalysts for thermocatalytic CO_2_ conversion into syngas and fuels

## 1. Introduction

Global energy-related CO_2_ emissions are heading for their second-largest annual increase ever. Demand for all fossil fuels was set to grow significantly from 2021. Coal demand alone is projected to increase by 60% more than all renewables combined, underpinning a rise in emissions of almost 5% or 1500 Mt. This expected increase would reverse 80% of the drop in 2020, with emissions reaching 1.2% (or 400 Mt) below 2019 emissions levels [1] The CO_2_ emission rate has continued to grow, which is expected to elevate the Earth’s temperature into a new elevated level without intervention. The global dependency on fossil fuel as feedstocks has contributed to most CO_2_ emissions [2,3]. Not to lose a sight of fact, natural resources of CO_2_, which can come from ocean degassing, plant photosynthesis, and volcanic eruptions, are reviewed as a natural sink contributing toward rapid atmospheric CO_2_ build-up. The massive ocean sink has dramatically affected ocean climate change and ecosystem [4]. On top of all the factors is the human factor, characterized by extensive burning of fossil fuels, which has worsen climate change and created an ocean acidification phenomenon. Coral bleaching and destruction of coral reef structures are the aftermath. Hence, novel renewable and sustainable energy sources are needed to protect the environment and ecosystem [5]. CO_2_ fixation has attracted broad interest, and intensive efforts have been dedicated to developing various technologies for CO_2_ capture, sequestration, and utilization [6]. As mentioned, CO_2_ as feedstock for chemical processes has attracted great attention because it can reduce the cost and increase the profit for reducing CO_2_ emissions. Acidic gases allow the introduction of a closed carbon cycle, which is important to achieve a circular economy.

**Figure 1:**
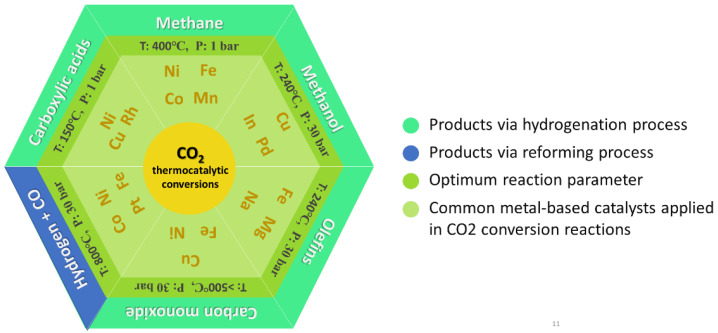
Illustration of CO_2_ thermocatalytic conversions into alternative fuels and gasoline via catalytic hydrogenation (denoted in green) and via reforming process (denoted in blue), and the corresponding typical metal-based catalysts that optimize the reaction performances.

Figure 1 briefly summarizes common thermally driven CO_2_ valorization, which involves the presence of metal catalysts. CO_2_ conversion into specialty chemicals, such as urea and salicylic acid, is performed using mature and well-established technologies [1,2]. However, the production scales are low, and the effect on global CO_2_ emissions is negligible. CO_2_ can be converted into hydrocarbon or other liquid fuels using direct or indirect routes, such as syngas production [3]. Catalytic reduction of CO_2_ by thermocatalytic (TC) hydrogenation is a feasible process to restrain the greatest CO_2_ emissions under an economic perspective. This acidic gas is used to reform hydrocarbons from natural gas or shale gas indirectly through dry CO_2_ reforming of methane (DRM). The direct route for CO_2_ conversion into fuel involves the reaction of CO_2_ with hydrogen to form CO, methane [5], methanol [6], olefins [7] and dimethyl ether (DME), depending on the catalyst and operation parameters (pressure, temperature, and reactor). CO_2_ hydrogenation can be performed by utilizing green H_2_ resources in the presence of efficient catalysts to afford value-added fuels and energy carriers. Hydrogenation is the most critical reaction in the industry because it is thermodynamically unfavorable and can only be performed under extreme conditions, such as high pressures and extremely high temperatures. Homogeneous catalytic systems have become an alternative; nonetheless, their large-scale use in the industry is restrained by chemical process challenges such as product separation and catalyst recyclability [8]. Therefore, heterogeneous catalytic systems for CO_2_ conversion remain the best option, especially thermocatalysis, which is likely to be favored as a research hotspot.

Current research on the chemical transformations of CO_2_ focuses on electrocatalysis, photocatalysis, and thermocatalysis for CO_2_ conversion [9,10,11,12,13,14,15,16]. Different catalytic systems attain different benefit perspectives in upscaling the application for commercialization purposes and optimal operation feasibilities, where the TC conversion of CO_2_ is desirable for large-scale applications with the advantages of simple process equipment, low cost, and easy industrialization enlargement. Although TC CO_2_ conversion is expected to reduce atmospheric CO_2_ concentration, the feasibility of net reduction is disputable. Most thermal CO_2_ hydrogenation processes require large energy input even with optimally designed catalysts, which cause concern toward the decarbonization target because 70% of products are yielded by CO_2_-emitting fossil hydrocarbon at present. From the 62 billion tons emitted CO_2_ by 2050, the International Energy Agency recommended that 48 billion tons of CO_2_ should be captured and utilized through various CO_2_ recycling technologies to limit the temperature increase to within 2 °C by that same year.

CO_2_ conversion catalysts with more intricate structures than conventional heterogeneous catalysts need to be discovered. Conventional solid catalysts traditionally consist of an active phase dispersed on stable porous support [13,17] with high surface area or promotion by additional metal additives to optimize the metal–support integration. However, this catalyst design does not provide much control over its structure at the atomic scale, thus resulting in poor active phase dispersion on the catalyst surface and low product selectivity, activity, and catalyst stability. Recent advances in nanomaterials and synthesis methods have remarkably improved the nanoparticle (NP) size, shapes, morphologies, and physicochemical tunability of catalysts for effective CO_2_ conversion. In past years, the rapid advancement in nanotechnology opened a novel prospect for application of advanced nanomaterials as catalysts for several industrial processes. Core-shell nanostructures (CSNs) are now at the core of progress in CO_2_ conversion catalysis, where spherical NPs provide a controlled integration of various components and exhibit multifunctional properties. Despite the aforementioned potential, the utilization of nanomaterial-based catalysts in scalable industrial applications remains challenging because the mechanisms by which these nanomaterials efficiently facilitate different industrial processes are poorly understood [14].

Previous reviews focused on the roles of CSN catalysts in photocatalytic [18] and electrocatalytic [19,20,21,22,23]. CO_2_ conversions, including CO_2_ electroreduction, CO_2_ electrooxidation, water splitting, and CO_2_ reforming, but only a few have summarized their roles in TC CO_2_ hydrogenation [5]. Those reviews provide abundant insights into the activation of CO_2_ and formation of syngas and synthetic alcohol derivatives. Some works also concentrated on catalytic systems, thermodynamic challenges in CO_2_ TC conversion, and roles of transition metal-based heterogeneous catalysts as frameworks, but few discussed the physicochemical effects and mechanisms of CSN catalysts, fabrication methods, and reaction conditions for CO_2_ TC conversions. Thus, the present review provides a comprehensive and systematic discussion on such aspects by summarizing recent studies on CO_2_ TC conversion using CSN catalysts. The performance of nanocatalysts in several industrial CO_2_ hydrogenation reactions for methanol synthesis and methanation are comprehensively discussed. An alternative route to utilize CO_2_ by reforming the gases into syngas is also discussed by presenting recent works on CSNs over CO_2_ DRM.

We focus on the unique functionalities, applications, and benefits of CSNs in CO_2_ TC conversion. This review is grouped into several sections: introduction, CSN strategies for thermochemical CO_2_ catalysis, and applications of CSNs in CO_2_ utilization reactions, specifically CO_2_ hydrogenation to methane, CO_2_ hydrogenation to methanol, and CO_2_ dry reforming of methane.

## 2. Approaches to the Synthesis of Core-Shell Catalysts for CO_2_ Utilization

Das et al. have compiled several applications of CSNs to tackle the challenges faced by various CO_2_ conversion processes [3]. Recently, concerning electrocatalytic [19,20,22,24,25,26] and photocatalytic [27,28,29,30,31,32]. CO_2_ conversion processes, special attention has been focused on the use of these nanostructured catalysts as alternatives to conventional heterogeneous catalysts. Among the three mentioned applications, CSN catalysts have emerged the most in electrocatalytic applications, such as fuel cells, batteries, supercapacitors, and electrolysis [33]. Given the combined benefits of shell and core components, CSNs have been applied as cathodes to protect the layers and overcome the deficiencies of the original cathode. Core-shell catalysts for CO_2_ TC conversions remain less popular than the conventional catalysts in the field. However, those conventional catalysts for CO_2_ conversion remain to be upscaled.

Surface and interface engineering of catalysts is important in catalysis. The selectivity and stability of catalysts serve as pivotal concepts, and rapid deactivation is a major challenge affecting the overall performance of reactions, catalytic activities, and product selectivity [3,34,35]. These parameters depend on the atomic structure of active sites on the surface [36,37,38,39,40,41]. Catalyst deactivation mechanisms are often categorized as sintering, poisoning, poor defects, fouling, and leaching on the catalyst surface [42,43] Sintering of active metals usually occurs when catalysts endure high temperatures during catalyst preparation or CO_2_ activation. Since CO_2_ conversion thermodynamically requires high temperatures to achieve economically viable conversions of the reactants, the catalyst NPs may bond between atoms and crystallites, causing the broken surface structure and agglomeration and leading to catalyst deactivation [44]. Various strategies to prevent deactivation have been adapted. One of these strategies is converting the catalyst structure to CSNs [17,45].

**Figure 2:**
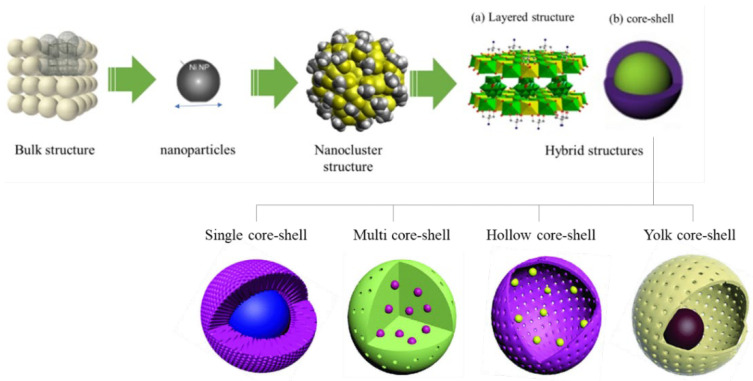
Evolution of catalyst surface engineering, which plays a role in catalyst design strategies: (**a**) layered double hydroxide (LDH) and (**b**) core-shell as new and promising catalyst structures for enhancement of thermocatalytic CO_2_ reactions. Illustration of different types of core-shell with different multifunctionalities is reproduced from Das et al. [4], Core-shell structured catalysts for thermocatalytic, photocatalytic, and electrocatalytic conversion of CO_2_; published by the Royal Society of Chemistry, 2020.

Over decades, catalyst engineering has evolved into different structures, from bulk metal structures to NP, nanocluster, and hybrid structures (core-shell, layered interface) (Figure 2) [46]. The structural transformation of catalysts provides active sites that affect the surface reaction mechanism of CO_2_ conversions. Hybrid structures have rapidly advanced by the breakthrough of CSNs. The term ‘core-shell’ was first proposed in early 1990 when a group of researchers synthesized a multilayer semiconductor NPs [47] This unique catalyst is composed of a core as the inner material and a shell as the outer layer. As a type of core-shell component, composite CSNs, in which the CSN materials contain different ingredients, have been widely explored in terms of their synthesis [48] and applications [42,49,50,51] The morphologies of conventional supported catalysts have been described as active particles that are layered or exposed on top of porous supports [52,53] Meanwhile, CSNs inherit spherical-like particles that can be encapsulated by shell porous materials [13]. The robust shells afford 360° protection for active cores during catalyst preparation and TC reaction, which enable high exposure of catalytic sites and thus high performance in long-term reactions [54]. Encapsulation is introduced to confine the growth of core particles that are easily sintered or migrated during fabrication at extremely high temperatures. Two components acting as core and shell can modulate the interface by providing strong metal-support interactions (SMSIs), which regulate the surface electronic properties of active sites. In catalysis, a porous shell allows reactants to be carried into the core and produce selective products from the core [13].

**Figure 3:**
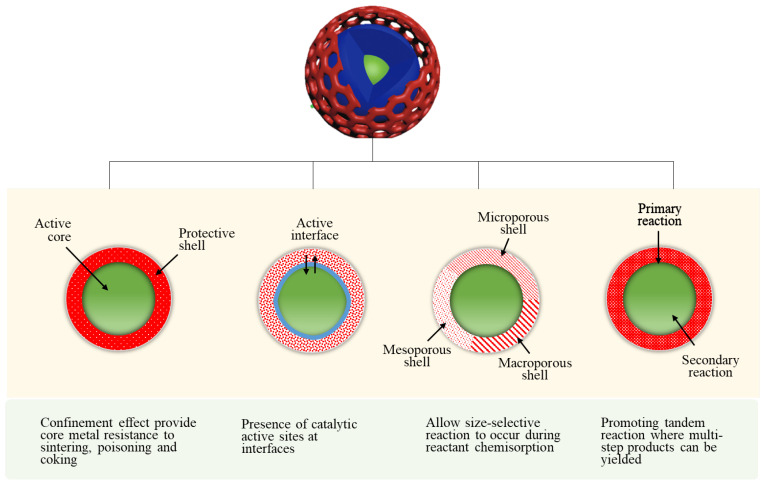
Partial schematic of core-shell structure strategies and significance properties in thermocatalytic CO_2_ conversion. Reproduced illustration with permission from Das et al. [3], Core-shell structured catalysts for thermocatalytic, photocatalytic, and electrocatalytic conversion of CO_2_; published by the Royal Society of Chemistry, 2020.

CSN catalysts have functional benefits in heterogeneous catalytic reactions, as shown in Figure 3. The core-shell confinement exerts positive influence on reaction selectivity, reactivity, and stability. Particle geometric and spatial distribution, structural homogeneity, interfacial effect, sintering rate, and product selectivity are factors that determine the stability and selectivity of CSNs in TC application. Regardless, some strategies must be employed before these CSNs can offer such benefits.

Commercial use of conventional heterogeneous catalysts is limited by their poor stability in reaction conditions. Although commercial heterogeneous catalysts consist of well-dispersed metal species stabilized on high-surface-area supporters and are cost effective, their nanostructures are poorly controlled and cause rapid instability. Therefore, in recent years, fruitful efforts to understand the fundamental issue have been made by researchers in this niche, as evidenced by the increasing number of publications devoted to overturn catalytic deactivation. Carbon deposition, metal sintering, thermodynamic instability, and catalyst fouling are the main reasons for catalyst deactivation in CO_2_ thermodynamic conversion [55]. CO_2_ DRM reaction results in the formation of two main forms of carbon deposit: encapsulating and filamentous coke. Meanwhile, sintering and fouling mainly occur in CO_2_ hydrogenation reactions due to thermal instability.

CSN catalysts help eradicate such deactivation by preventing the rapid inactivation and deposition of active metals as sintered metals on the catalyst surface. The size of metal crystallite can be controlled when the core-shell structural arrangement is tailored by encapsulation, thereby restraining the degree of agglomeration, which improves the stability of catalysts in reaction over conventional catalysts. Sintering usually occurs at high reaction temperatures and pressures, in the presence of steam or weak metal-support interactions. Sintering exerts an adverse effect on the number of exposed metal active sites on the catalyst surface, leading to reduced catalytic activity.

Almost all CO_2_ conversion processes apply the thermodynamic concept dictating that the conversion of nonoxidative CO_2_ and other reactants to high-selective products can only be achieved at very high temperatures. Therefore, selection of supports with high thermal resistance and structural porous channels are crucial to lower the reaction temperature while ensuring superior conversion rate and selectivity [56] Surface tuning via core-shell structure and porosity can induce a nanoconfinement effect, which increases the probability for CO_2_ or intermediates to interact with CSN catalysts, thereby enhancing CO_2_ adsorption and conversion. CSN catalysts with mesopore (2–50 nm) or nanopore (<2 nm) size distributions can trap selected reaction intermediates and CO_2_ molecules, prolonging the residence time inside the channels. Such catalysts increase the CO_2_ concentration within the shell pore channels and promote CO_2_ conversion into useful derivative products. For example, Jiao et al. fabricated a confined Cu@In_2_O_3_ CSN catalyst with In_2_O_3_ pore size distribution of 100–200 nm. The complete dominancy of In_2_O_3_ porous channels over the Cu NPs significantly improves the reactant transport across the core and shell interfaces during diffusion [57].

In addition to trapping selectively reactant-size molecules, the confined structure provided as core-shell may also influence mass transport and charge transfer. More multilevel channels are constructed from the innermost active sites. Hence, CO_2_ molecules and reaction intermediates can transport rapidly via the interconnected channels, facilitating the dissipation of products and replenishing the fresh reactants. CSNs establish an opportunity for catalyst design to achieve selective production from CO_2_ conversions. The production capacity yielded by CO_2_ gas is a crucial index to evaluate the development of the petrochemical industry, as most products (CH_4_, CH_3_OH, and olefins) are building blocks for a wide range of chemicals. However, its industrial production is highly energy intensive, which involves high temperature and infeasible distillation. Some direct CO_2_ conversion processes are advantageous alternatives, but they still can suffer from poor production rates [58,59].

The nanoconfinement effect of core-shell catalysts enhances the catalytic activity and reaction pathway by changing the local pH around the catalyst surface. Intrinsically, the surface porosity and cavities could change the retention times of the intermediates, thereby influencing the reaction pathway. These observations imply that the core-shell structure can greatly affect mass transport, thus altering the reaction environment within the pores. The key to a functional catalytic structure is the rational design for nanocomposite growth sequences. For core-shell catalysts to exhibit such functionalities, uniform growth must be precisely controlled as outlayer material (shell) on the surface of the inner material (core). Therefore, the synthesis of core-shell catalysts is crucial and slightly different from that for conventional heterogeneous catalysts. In general, impregnation of heterogeneous catalysts on supported metal oxides leads to the stochastic distribution of the size and spatial location of the active NPs within the catalyst volume [60]. Additionally, CSNs can be engineered via additional synthesis following hydrothermal [61,62,63] microemulsion [64,65,66,67], ball milling [68,69] or ion exchange [70,71] technique. Such additional synthesis steps are important to tailor the water-to-surfactant ratio, thus resulting in a small and narrow particle size distribution inside the shell cage.

Hu et al. [72] reported that CSN growth can differ depending on the synthesis approach used. For instance, nondestructive methods allow the formation of a core-shell structure without destroying the lattice structure, leading to the formation of a hollow chamber in between the core and shell interfaces. These methods provide an ideal environment for core crystal growth and allow for the generation of the amorphous phase of the shell. By contrast, destructive methods produce a shell with a partially destroyed lattice structure, leading to the generation of an amorphous shell covering the well-crystallized core. Hydrothermal and solvothermal methods are categorized as nondestructive approaches to create a core-shell structure. The formation of core-shell nanocomposites is constructed on the reaction between the pre-grown porous shell materials and metal core precursors. The thickness and morphology can be modulated by controlling the molar ratio of core-shell composition. Some cases introduce a structure-directing agent to guide the synthesis or act as a template for shell formation, whereas others do not employ a template. Moreover, the deposition time and reaction temperature during synthesis can affect the growth of core-shell nanocomposites. Adequate time allows the shell materials to be facilely encapsulated on the core nanocrystal. A breakthrough synthesis method, such as ion exchange, has been used to convert one crystalline solid into another. This method generally follows the cation exchange route [47]. However, the growth of the core-shell structure via this method is hard to control.

CSNs can be functionally designed catering to specific needs. High temperature stability is usually enhanced when the active sites are designed at the core part and embedded within the shell because the strong interaction between the active sites and the surrounding shell materials retards sintering growth. Catalytic activity can be improved by modifying shell porosity and thickness, optimizing the size of active metal particles with shell protection, and maximizing the synergistic effect between the active metal and shell material. High-quality catalysts that fit certain application should be able to provide active sites that dissociate H_2_ without binding CO_2_ molecules significantly to prevent poisoning [8]. Core-shell confinement leads to a high local concentration of CO_2_, intermediates, and products at the active sites, resulting in high reaction rates [73] Interestingly, the confined core-shell particles show better reactivity and selectivity than the NPs externally decorated on the surface of the support catalyst in some essential CO_2_ conversion processes. For instance, in CO_2_ methanation, the encapsulated Ni NPs inside a CeO_2_ shell [74] depict better particle size control and spatial distribution (~7 nm) than the conventionally prepared Ni/CeO_2_ catalyst (~15.5 nm) [75].

The selection of core and shell materials depends strongly on the end application and use. Properties such as reactivity or thermal stability can be modified by changing either the constituting materials or the core-to-shell ratio so that they exhibit unique properties. The purpose of core particle encapsulation internally is to increase the functionality and surface modifications, minimize the usage of precious metals, and improve the stability and dispersibility of active metals. The stability of active NPs toward sintering may improve owing to the strong interaction between active metals and functionalized support interfaces, as illustrated in Figure 2. Similarly, Price et al. [76] confirmed that the confinement effect on the active sites could avoid sintering and that the composition of metal and shell materials has the potential to rapidly functionalize the shell porous channels with hydroxyl (^−^OH) groups via CO_2_ molecular dissociation, which can be summarized as highly effective in coke deposition and particle agglomeration prevention.

## 3. CO_2_ Conversion Processes and Products

Methanol, cyclic carbonates, dimethyl carbonates, isocyanate, carboxylic acid, hydrocarbons, CO, and olefins are valuable common industrial products converted via CO_2_ catalytic conversion [54]. Inspired by those homogeneous and heterogeneous catalytic systems, increasing attention has been given to the design synthesis and evaluation of reticular frameworks and their derived materials for CO_2_ capture and conversion because of their unprecedented porosities with CO_2_-philic groups and active centers [8]. The thermocatalysis of CO_2_ into fine chemicals can be conducted via the hydrogenation of CO_2_ to chemicals and fuels as CO_2_ molecules dissociate by activated hydrogen atoms in hydrogenation, which requires hash conditions (low temperature and pressure).

In general, the catalytic conversion of CO_2_ into added-value chemicals and syngas can be affected by many factors. For instance, a high density of active sites leads to enhanced activity and efficiency for CO_2_ conversion. Catalysts with high density of active sites, high surface areas, and large pore sizes are essential for efficient mass diffusion. In addition, increasing the CO_2_ pressure might facilitate the conversion by increasing CO_2_ concentration or reducing the melting point of organic compounds [4]. However, high CO_2_ pressures may also decrease the yield by forming two phases, which are associated with contact problems between the substrate and the catalyst, and disrupt the catalyst’s composition. High temperatures enhance the reaction rate but may decrease selectivity, especially for enantioselective catalytic systems.

### 3.1. CO_2_ Hydrogenation Reactions

In general, CO_2_ conversion can be achieved using a multitude of reactions. In CO_2_ hydrogenation, these reactions include the reverse water–gas shift (RWGS) (Equation (1)), Sabatier reaction (Equation (2)), CO_2_-to-methanol (Equation (3)), and Fischer–Tropsch synthesis (Equation (4)) [77,78]:

(1) $${\mathrm{CO}}_{2}+{\;H}_{2}\;\underset{}{↔}\;\mathrm{CO}\;+{\;H}_{2}O\;;\;\Delta H_{298K}=41.2\;\mathrm{kJ}/\mathrm{mol}$$

(2) $${\mathrm{CO}}_{2}+4H_{2}\;\underset{}{↔}{\;\mathrm{CH}}_{4}+{\;H}_{2}O\;;\;\Delta H_{298K}=-165\;\mathrm{kJ}/\mathrm{mol}$$

(3) $${\mathrm{CO}}_{2}+3H_{2}\;\underset{}{↔}{\;\mathrm{CH}}_{3}\mathrm{OH}\;+{\;H}_{2}O\;;\;\Delta H_{298K}=-49\;\mathrm{kJ}/\mathrm{mol}$$

The RWGS reaction has become a research hotspot owing to its capability toward CO_2_ recycling and production of 1:1 syngas. This reaction is the key intermediate step in any CO_2_ hydrogenation because it effectively produces CO from CO_2_. It consumes H_2_ and produces CO, which is much more reactive as a feedstock for C1 chemistry than CO_2_ molecules with carbon double bonds, thereby facilitating the reaction at low temperatures. Equations (3) and (4), which undergo CAMERE and FT processes, enhance catalytic efficiencies when the CO generated from RWGS reactions Equation (1) is used as the raw feedstock.

(4) $$n\mathrm{CO}+\left(2n+1\right)H_{2}\;\underset{}{\to }C_{n}H_{2n+2}+nH_{2}O;\;\;olefins\;\;production$$

#### 3.1.1. CO_2_ Methanation

CO_2_ methanation, a process of methane production via CO_2_ hydrogenation, is a pioneering technology that has received extensive recognition for providing solutions toward CO_2_ emission reduction, renewable energy utilization, and natural gas market reliever [79]. Abundant greenhouse gases (GHGs) in the atmosphere can be utilized to yield clean and green fuels via CO_2_ methanation. Methane (CH_4_) is the main natural gas formed with higher combustion value, and its combustion products are clean and safe relative to other fossil fuels. CH_4_ has high energy density and is easy to store, making it an efficient renewable hydrogen energy carrier.

The Sabatier reaction (Equation (2)) is a highly exothermic reaction that only occurs completely at low temperatures between 423 K and 573 K and high atmospheric pressure (1 atm) in the presence of a catalyst [72] to obtain optimum CO_2_ conversion and CH_4_ selectivity. However, the standard heat of CO_2_ formation is −394.38 kJ/mol, and its high chemical inertness suppresses its activation and molecular dissociation. In addition, at temperatures above 300 °C, RWGS takes over the activity, thereby increasing CO selectivity, decreasing CH_4_ selectivity, and promoting CO_2_ conversion.

(5) $$2{\mathrm{CO}}_{\left(g\right)}↔{\mathrm{CO}}_{2\left(g\right)}+{\;C}_{\left(s\right)};\;\Delta H_{298K}=-171\;\mathrm{kJ}/\mathrm{mol}$$

Similar to other CO_2_ hydrogenation reactions, methanation faces challenges, such as catalyst deactivation due to carbon deposition (fouling, Equation (5)) and decreased activity, resulting in a short catalyst lifetime. In order to prolong the catalyst lifespan and prevent catalyst degradation, CO_2_ methanation catalysis has received huge attention to develop high-activity catalysts at low temperatures [5]. Gao et al. designed an adequate ratio of CO_2_/H_2_ and found that a sufficient amount of H_2_ can significantly influence the production of water vapor (H_2_O), increase methanation activity rather than CO formation, and inhibit carbon deposition.

Various catalyst preparation methods, including conventional impregnation, sol–gel, and coprecipitation methods, have been developed to discover ways to achieve high activity and selectivity under mild conditions, good stability, and service life of CO_2_ methanation catalysts. Therefore, suitable active metals, such as Cu, Ni, Fe, Pd, and Co, have been reported for CO_2_ methanation, and Ni-based catalysts have been selected for industrial commercialization. However, these conventional metal-based catalysts often show low reaction activity at low temperature (180–300 °C) and tend to form particle agglomeration and sintering at high temperatures. For instance, Ni/Al_2_O_3_ catalysts prepared by wetness impregnation exhibit low metal dispersion and sinter in the presence of water at high temperatures, thereby increasing CO selectivity and energy consumption. Thus, active Ni metal with low loading is weakly dispersed and becomes less active at low temperatures, whereas the catalyst deactivates at high temperatures due to sintering. Catalyst design has been improved by adding promoters (Fe, Pd, Rh), additives, and support metal oxides (SiO_2_, ZrO_2_, CeO_2_) to enhance activity at low temperatures. However, addition of these components in the conventional method is detrimental toward efficient CO_2_ adsorption.

##### Core-Shell Nanostructured Catalysts for CO_2_ Methanation

**Figure 4:**
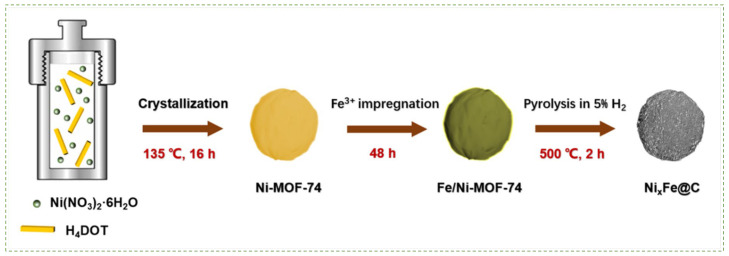
Schematic of the synthesis of the Ni_7_Fe@C catalyst by pyrolyzing Ni-MOF-74. Reproduced with permission from Li et al. [77], Iron promoted MOF-derived carbon encapsulated NiFe alloy nanoparticles core-shell catalyst for CO_2_ methanation; published by Elsevier, 2022.

Core-shell confinement of structure has garnered interest as a potential nanostructure support for CO_2_ methanation. The activity and selectivity of core-shell structured catalysts are mainly affected by the type of metal used. Among porous shell supports [13], metal-organic frameworks (MOFs) are a potential porous shell for Ni confinement because of their porous crystalline materials, high specific surface area, and tunable uniform elemental distribution. As shown in Figure 4, Li et al. [77] prepared a Ni_7_Fe@C core-shell catalyst by pyrolyzing Ni-MOF-74 for low-temperature CO_2_ methanation. Ni precursors were treated solvothermally in a solvent solution at 136 °C to induce crystallization, and the Ni-MOF-74 suspension is calcined and then reduced in 5% hydrogen to form the Ni_x_Fe@C catalyst. The obtained catalyst achieves a CO_2_ conversion of 72.3% with 99.3% CH_4_ selectivity at 350 °C. Encapsulation of Ni-Fe alloy within carbon porous structure facilitates high CO_2_ adsorption and effectively prevents the aggregation of active metal NPs during the reaction, thereby conferring the core-shell catalyst with superior stability. Moreover, the homogeneity of Ni-Fe NP elemental distribution can be preserved, which improves Ni dispersion.

New core-shell nanostructure based on cobalt (Co) catalysts have been successfully fabricated by Cui et al. [78] to study catalytic performance of low temperature methanation. MnO-heterostructured NPs injected into porous graphitic carbon (Co/MnO@PGC) were synthesized via a single-step pyrolysis of bimetal CoMn@MOF-74. The resulting nanocomposite features an enriched Co/MnO heterointerface and exhibits excellent catalytic performance for low-temperature CO_2_ methanation. The synthesized Co/MnO@PGC catalyst allowed CO_2_ molecules to activate faster at a low heat of 160 °C over 99% selectivity with high STY_CH4_ of 0.14 μmolCH_4_⋅s ^−1^ gcat^−1^. As the temperature reached 240 °C, CO_2_ conversion and space–time yield (STY_CH4_) rose to 32.1% and 13.34 μmol_CH4_⋅gcat^−1^⋅s^−1^, respectively. At a high pressure (30 bar), STY_CH4_ can reach up to 5.60 μmol_CH4_⋅s^−1^⋅gcat^−1^ at 160 °C, which is even comparable to that of the optimal level of Ru-based catalysts. These results indicate that the synergistic interactions between Co and MnO NPs at the Co-MnO heterointerface are responsible for enhancing the catalytic activity toward CH_4_ production at a low temperature. In addition, the Co/MnO heterostructured NPs encapsulated into PGC play an important role in preventing metal particle aggregation and improving thermal stability. High TOF_CH4_ suggested that the Co/MnO heterointerface formed inside the PGC of Co/MnO@PGC can significantly boost its activity of low-temperature (160–220 °C) CO_2_ methanation.

Core-shell metal@metal oxide particles can be promising as high thermal-conducting support materials owing to the high thermal conductivity of metal and excellent surface structural properties of the metal oxide itself. Various supports, such as Al_2_O_3_ [80], CeO_2_ [81], ZrO_2_, SiO_2_ [60], or zeolites [60] have been proposed as metal oxide shell to protect the active metals at the core. In addition, Le et al. [82] synthesized a Ni/Al@Al_2_O_3_ CSN catalyst for CO and CO_2_ methanation by using hydrothermal surface oxidation (HTSO). Ni/Al@Al_2_O_3_ has selectively yielded carbonate and formate species, which suppress the CO intermediate. The confinement effect helped the CSN catalyst lower the activation energy barriers (74 kJ/mol), which outperforms the activation energy of conventional catalysts, namely, Ni/Al_2_O_3_ (80 kJ/mol) and Ni/SiO_2_ (89 kJ/mol). Apparently, Ni/Al@Al_2_O_3_ CSN can successfully enhance the catalytic CO_2_ adsorption owing to its high Ni dispersion and strong CO_2_ binding.

**Figure 5:**
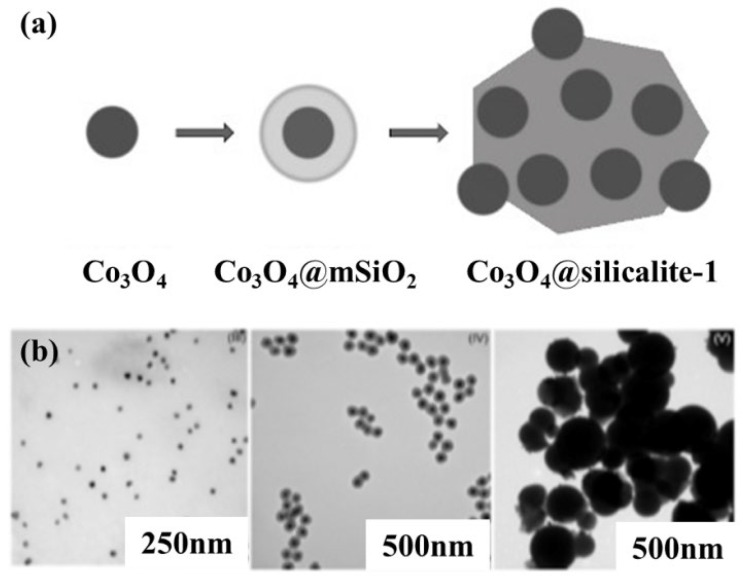
(**a**) Schematic bottom-up synthesis of Co@SiO_2_ and Co@Silicalite-1 via a solvothermal method and (**b**) morphological TEM images of Co@SiO_2_ and Co@Silicalite-1 (from left to right). Figure reproduced from Ilsemann et al. [60], Cobalt@Silica Core-shell Catalysts for Hydrogenation of CO/CO_2_ Mixtures to Methane; copyright from *ChemCatChem*, John Wiley and Sons, 2019.

Meanwhile, Ilsemann et al. [60] prepared Co@SiO_2_ and Co@Silicalite-1 catalysts via a solvothermal method to encapsulate the Co NPs inside two mesoporous structures of silica shells (Figure 5). They found that Co@SiO_2_ improves the catalytic activity in low-temperature CO_2_ methanation (230°C–400 °C) by suppressing the side reaction (RWGS), which results in highly selective CO_2_ hydrogenation to methane. The thermal stability provided by mesoporous silica could preserve the active Co metals at elevated temperatures. However, CO methanation causes slight coking, resulting in a shift of kinetic stability and reduction in methane yield. Similarly, a silicalite-1-confined Ni catalyst was prepared through the selective desilication of the molecular sieve to produce extra voids and pore channels to cage Ni in the crystal [83]. The Ni@Silicalite-1 catalyst is characterized by higher CO_2_ conversion and CH_4_ selectivity than conventional Ni/Silicalite, which can be attributed to the higher Ni fine dispersion in the void of silicate. The catalyst maintains stable performance over 50 h at 450 °C.

**Figure 6:**
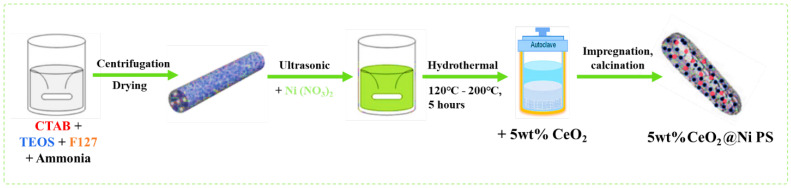
Schematic of formation of Ni-phyllosilicate catalysts via a hydrothermal method. Reproduced with permission from Yang, Zhang, and Liu [81], Highly Efficient Ni-Phyllosilicate Catalyst with Surface and Interface Confinement for CO_2_ and CO Methanation; copyright from American Chemical Society, 2021.

CSNs are expected to provide an anti-sintering effect by anchoring the active metal NPs in the porous channel. The formation of metal–metal oxide compounds modulates strong interaction to realize the low deactivation rate of reaction. However, this core-shell design has some shortcomings, such as complex preparation, expensive instrumentation, and lack of exposed defect surface, all of which restrict its industrial applications. Therefore, Yang et al. [81] prepared Ni-phyllosilicate@CeO_2_ CSN by using a hydrothermal method, as shown in Figure 6, to create Ni fine dispersion (3.3–6.3 nm). The anchored Ni phyllosilicate could further increase the H_2_ and CO_2_ uptake, contributing to high CO_2_ conversion rate (65%), thus exhibiting high catalytic activity and stability for 100 h lifetime CO_2_ methanation.

**Figure 7:**
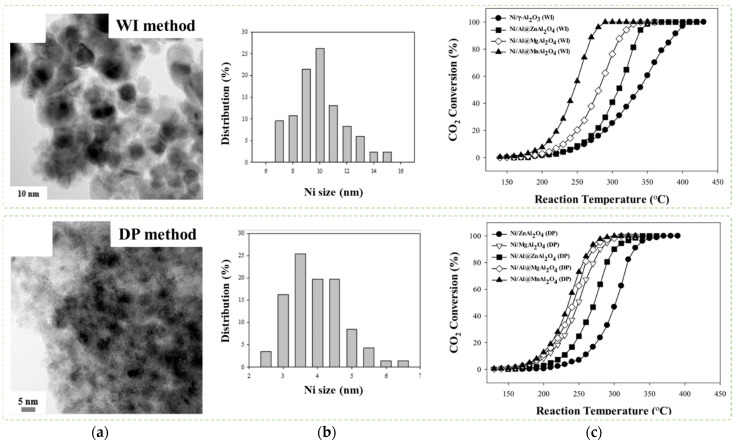
(**a**) HR-TEM image of Ni/Al@MnAl_2_O_4_ (WI) and Ni/Al@MnAl_2_O_4_ (DP), (**b**) Ni particle size distribution in Ni/Al@MnAl_2_O_4_ (WI) and Ni/Al@MnAl_2_O_4_ (DP), (**c**) catalytic performance of Ni/Al@MAl_2_O_4_ prepared using the WI and DP methods for CO_2_ methanation. All catalysts were reduced in H_2_ at 500 °C. Reaction conditions: 1 mol% CO/CO_2_, 50 mol% H_2,_ 49 mol% He, F/W = 1000 mL/min g.cat. Retrieved with permission from Le et al. [84]; CO_2_ Methanation over Ni/Al@MAl_2_O_4_ (M = Zn, Mg, or Mn) Catalysts; copyright from MDPI, Catalysts, 2019.

Le et al. [84] continued their work on the Ni/Al@M-Al_2_O_4_ core-shell catalyst by promoting various transition metals (M = Mg, Ni, Co, Zn, or Mn) to develop a synergistic interaction between M/Al and enhance the catalytic activity of the CSN in low-temperature CO_2_ methanation. Different influences of thermal conductivity on shell NP dispersion were observed as an anti-sintering property. Ni/Al@M-Al_2_O4 CSNs were prepared using deposition-precipitation (DP) and wet impregnation (WI) methods to facilitate superior heat conductivity and surface properties for highly exothermic and endothermic reactions and control the effect of parameters on metal particle size. Morphological analyses showed that 9 wt% Ni/Al@MnAl2O4 (DP) has a significant BET surface area of 129 m^2^/g and the highest Ni metal dispersion (9.7%) among other synthesized catalysts. Introduction of Mg into the spinel Ni/Al@MgAl_2_O_4_ CSN has provided a larger BET surface area of 171 m^2^/g with the same dispersion quality to Ni/Al@MnAl_2_O_4_. Ni/Al@MnAl_2_O_4_ (DP) and Ni/Al@MgAl_2_O_4_ (DP) have demonstrated better catalytic performance than the catalyst prepared using the WI method (refer to Figure 7). Both catalysts selectively produce high methane yield by hindering more chain of hydrocarbon while facilitating 90% CO_2_ conversion under 300 °C, thereby promoting high catalytic activity for CO_2_ methanation. Bimetallic Ni-Al has facilitated functional heat transfer across Ni/Al@M-Al_2_O_4_ CSN catalyst particulates as the Al metal releases high heat conductivity. Interestingly, Ni/Al@MnAl_2_O_4_ shows superior catalytic stability because it has a lifetime of 50 h while preventing coke deposition and Ni particle agglomeration during CO_2_ methanation.

**Figure 8:**
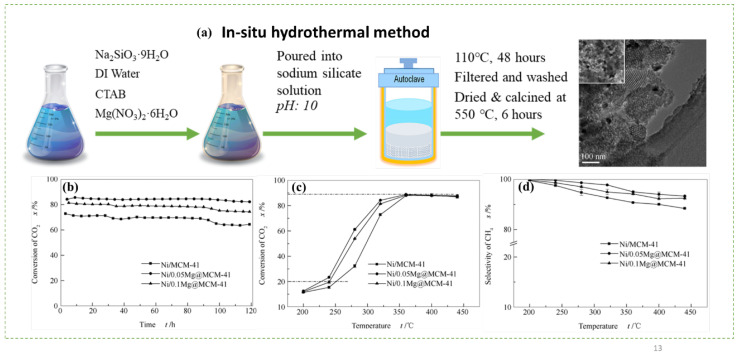
(**a**) Compilation of Ni/Mg@MCM-41 CSN synthesis by the in situ hydrothermal method and resultant SEM morphology of the core-shell structure formed, and the catalytic activities of Ni/MCM-41, Ni/0.05Mg@MCM-41, and Ni/0.1Mg@MCM-41, for comparison purposes between traditional and conventional CSN synthesis methods in terms of (**b**) stability of the catalysts in CO_2_ conversion at 320 h rather than 120 h; (**c**) catalytic conversion of CO_2_; and (**d**) selectivity of CH_4_. Retrieved with permission from Wang et al. [85] CO_2_ methanation over Ni/Mg@MCM-41 prepared by in situ synthesis method; copyright from Elsevier, 2020.

Considering the sharp rise in temperature of methanation and rapid catalyst deactivation by Ni particles, Wang et al. [85] suggested the interaction of Mg with Ni as a bimetallic core in a Ni/Mg@MCM-41 duo-core@shell catalyst prepared using an in situ hydrothermal method with different Mg contents. Wang et al. also synthesized a conventional Ni/MCM-41 by using WI for comparison of catalytic performance. They found that Ni/MCM-41 features a larger BET surface area of 622.5 m^2^/g than 0.05 wt% Ni/Mg@MCM-41, and the specific surface area continues to decrease as an additional 0.05 wt% Ni is incorporated. This result can be ascribed to the blockage of Mg particles on the pores when Mg^2+^ has less tendency to replace Si^4+^ ions in the SiO_2_ lattice. However, the Ni/Mg@MCM41 core-shell catalyst produces better atomic composition than the conventional Ni/MCM41 catalyst. Under an optimal temperature of 360 °C, 0.05 wt% Ni/Mg@MCM-41 exhibits the highest CO_2_ conversion at 88% during CO_2_ methanation (Figure 8). The selectivity of CH_4_ gradually decreases with increasing temperature, proving that high temperatures are not advantageous to CH_4_ production because CO_2_ methanation is an exothermic reaction. Regardless, the 0.05 wt% Ni/Mg@MCM-41 catalyst shows high catalytic activity at low temperatures (below 360 °C) and a large specific surface area (606.3 m^2^/g), which are suitable for CO_2_ methanation.

**Figure 9:**
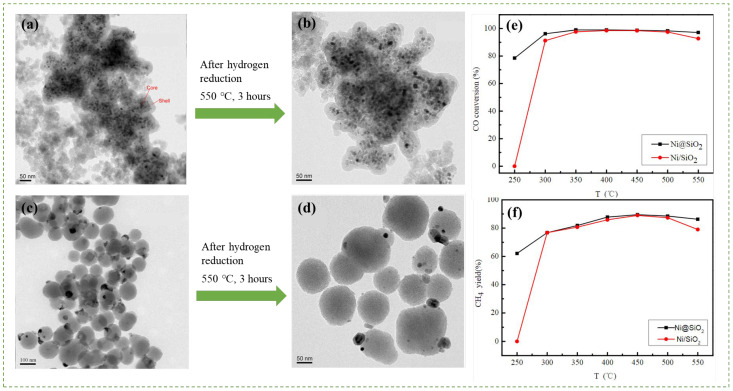
HR-TEM image of (**a**,**c**) fresh Ni@SiO_2_ and Ni/SiO_2_; (**b**,**d**) Ni@SiO_2_ and Ni/SiO_2_ after hydrogen treatment at 550 °C; (**e**,**f**) catalytic performance of Ni@SiO_2_ and Ni/SiO_2_ catalysts in CO methanation. Retrieved from Han et al. [86] Core-Shell Structured Ni@SiO_2_ Catalysts Exhibiting Excellent Catalytic Performance for Syngas Methanation Reactions; copyright from MDPI, *Catalysts*, 2017.

A well-defined nanostructured Ni@SiO_2_ core-shell catalyst (diameter size of 27.1 nm) was synthesized with distinct metal–metal oxide interfaces in proximity to each other to carry out CO_2_ hydrogenation [86]. Noteworthy, the Ni@SiO_2_ interface in the catalyst is responsible for RWGS reaction to form CO selectively. The strong interaction between Ni core and SiO_2_ shell effectively restrains NP growth (agglomeration) and carbon deposition. Thus, the Ni@SiO_2_ core-shell catalyst, as shown in Figure 9, yields 89.8% CH_4_ and successfully converts 99.0% of CO molecules. Moreover, it retains high catalytic stability in CO methanation under a 100 h lifetime condition, which surpasses the stability of the conventional Ni/SiO_2_ catalyst, whose CO conversion collapses after 12 h lifetime.

Ni@mpCeO_2_ CSN was synthesized using nanocasting, followed by strong electrostatic adsorption for CO_2_ methanation [74]. The turn of frequency (TOF) for the Ni/mpCeO_2_ CSN catalyst (0.183 s^−1^) at 225 °C is threefold higher than that of the Ni catalyst supported on conventional CeO_2_ prepared using the same method. Compared with the Ni catalyst, the Ni/mpCeO_2_ CSN provides a rich NiCeO_2_ interface with more oxygen vacancies, playing a key role in CO_2_ activation. CO_2_ activation over the Ni/mpCeO_2_ CSN catalyst occurs through combined associative and dissociative mechanisms that have been observed through DRIFT mechanism study. Ni NPs are highly dispersed in the channels of mpCeO_2_, which enhance H_2_ dissociation, thereby supplying sufficient *H species for the formation of CO and *HCO intermediate species owing to high CH_4_ selectivity. In addition to enhanced low-temperature activity and selectivity, the Ni/mpCeO_2_ catalyst maintains its stability 70 h on stream because Ni sintering has been suppressed by the confinement effect of mesoporous CeO_2_ structure. This study demonstrates the importance of the Ni-CeO_2_ interface, at which high oxygen vacancy concentration facilitates CO_2_ adsorption and activation while the adjacent Ni active sites accelerate H_2_ dissociation.

Table 1 summarizes the abovementioned literature reports on CSN application for CO_2_ hydrogenation to methane. Ni-based metallic catalysts remain the most promising for this reaction even for CSN. Previous researchers found that preparing CSN catalysts by solvothermal, in situ hydrothermal, or Stober methods promote the most well-defined CSN structure with better shell surface porosity, which is greater than that for the traditional prepared method. The key component to superior performance of catalytic hydrogenation reaction is its stability during the reaction period, how much main products it yields, and the selectivity or route it favors to yield the main products. Indeed, CSN catalysts have proved promising activities for these criteria.

#### 3.1.2. CO_2_ Hydrogenation to Methanol

Among all possible products in the CO_2_ hydrogenation reaction mentioned in the previous section, methanol (MeOH) is the most attractive (Equation (3)). MeOH is a clean, biodegradable, high-energy fuel and is highly versatile as it can easily generate other valuable fuels, such as DME, olefins, hydrocarbons, and long-chain alcohols [27,87,88]. Furthermore, combustion of MeOH generates few carbon side products owing to its freezing point (−96 °C). Thus, MeOH is suitable as a hydrogen carrier without producing a huge number of SO_x_ or NO_x_.

Unlike methanation, MeOH synthesis has thermodynamic and kinetic limitations, such as its high-pressure requirement for complete CO_2_ activation and low reaction temperature to selectively enhance the methanol yield [46,89] Nevertheless, this reaction still produces a competing side reaction, RWGS. Therefore, a potential route to suppress CO production must be discovered to maintain high methanol selectivity. CO_2_ hydrogenation to MeOH is favored at high pressures. Therefore, stable catalysts that are resilient to high temperatures and pressures are required. Active transition metals (Ni, Ru, Ga, Cu, and Co) are usually used for CO_2_ hydrogenation to MeOH because of their high activity at certain temperatures, abundance, low cost, and different oxidation states/phases to improve catalytic stability and selectivity. However, such active metals can encounter rapid deactivation (sintering, fouling, and poisoning) because methanol synthesis is a naturally structure-sensitive reaction, thus limiting CO_2_ activation.

Cu-based thermal catalysts are those used most often and hold many advantages for effective commercialization at the industrial scale. Cu catalysts are strongly active toward high methanol selectivity because of their three oxidation states (Cu^0^, Cu^1+^, and Cu^2+^) [90,91,92]. Cu provides active sites for H_2_ dissociation, and metal oxides increase the number of active sites for CO_2_ activation. Wang et al. reported that methanol selectivity corresponds to the proportion of strong basic sites to the total basic sites. Despite their benefits, Cu NPs agglomerate into large particles at elevated temperatures, which decrease MeOH yield. Therefore, understanding the changes in different surface atom arrangements on Cu NPs is important because the homogeneity of the catalyst structure affects the catalytic activity.

##### Core-Shell Nanostructured Catalyst for CO_2_ Hydrogenation to Methanol

CSN catalysts are attractive for these conversions because of the impact of the shell materials on reaction selectivity and catalyst stability [93] The core-shell nanostructure and surface have many advantages, such as enhancement of the essential properties of conventional catalysts. For example, commercialized Cu/ZnO/Al_2_O_3_ catalysts are unstable under high partial pressure at elevated temperatures, accelerating steam in the reaction atmosphere, hence causing rapid agglomeration and undesired crystal growth [3,27,94,95] A core-shell arrangement may optimize the interaction between the metal and porous support and minimize Cu sintering by creating unique multifunctionalities of catalytic sites.

An et al. [96] investigated Cu NPs coated with Zn to boost the surface electronic concentration and surface adsorption from high bimetallic synergy. The Cu/Zn bimetallic particles were anchored within the MOF network, and CuZn@UiO-bpy CSN was suggested to enhance the active metal dispersion and the SMSI. Cu NPs form within a diameter of 0.5–2 nm, depicting that the NPs are homogeneously dispersed, thus spreading more active sites and catalytic activity. Such structural properties result in remarkable CO_2_ conversion (17.4%) and methanol selectivity (85.6%). In addition, CuZnO@UiO-bpy CSN shows three times higher methanol yield at 250 °C than the conventional catalyst. This report agrees with the findings of Tisseraud et al.’s compilation study [97,98,99] where Cu@ZnO_x_ CSN catalysts exhibit 100% methanol selectivity as the oxygen deficiency formed by Zn migration provides active sites and hinders CO formation by side reactions (RWGS or MeOH decomposition).

**Figure 10:**
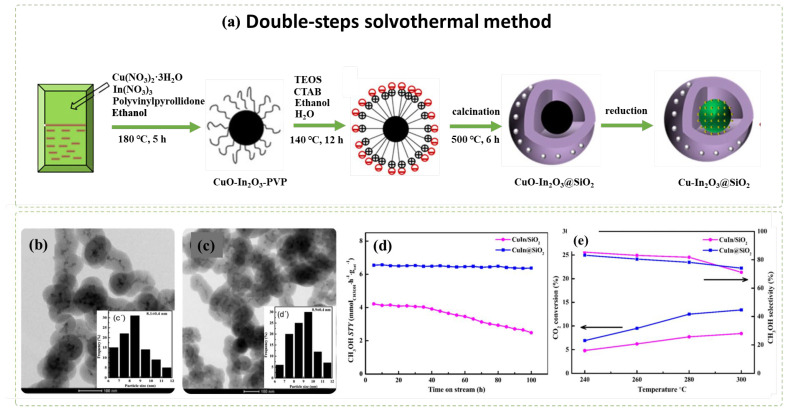
(**a**) CuIn@SiO_2_ synthesis by double-step solvothermal methods following its catalytic performance in methanol synthesis: (**b**,**c**) TEM morphological images of formed CuIn@SiO_2_ and reduced CuIn@SiO_2_, respectively; (**d**) space-time yield of MeOH over the catalyst under time-on-stream of 100 h; (**e**) CO_2_ conversion rates and product selectivity between conventional CuIn/SiO_2_ with CuIn@SiO_2_ core-shell catalysts. Reproduced with permission from the work of Shi et al. [57], A novel Core-shell structured CuIn@SiO_2_ catalyst for CO_2_ hydrogenation to methanol; copyright from *AlChE Journal*, John Wiley and Sons, 2018.

A recent study has prepared a CuIn@mSiO_2_ core-shell catalyst using a two-step solvothermal synthesis [57]. This catalyst was compared internally with Cu@SiO_2_, In@SiO_2_, and conventionally prepared CuIn/SiO_2_ catalysts. A perfect core-shell shape has been successfully synthesized as shown in Figure 10. The activity results showed that CuIn@SiO_2_ outperforms the others in terms of CO_2_ conversion but exhibits the second lowest methanol selectivity (21.8%) owing to CO formation at 250 °C. Nevertheless, the most stable performance over 100 h is dominated by Cu@SiO_2_ with zero sign of carbon deposits. Considering that mesoporous silica (mSiO_2_) shells are highly effective in limiting metal agglomeration and preserving the original metal particle sizes by providing a layer of thermally stable surface, Yang et al. [100] embedded Cu/ZnO within a layer of mSiO_2_ and achieved stable CO_2_ conversion and methanol yield over 160 h lifetime at a low temperature (260 °C). They observed that Cu/ZnO@mSiO_2_ shows better catalytic performance than the conventional impregnated catalyst Cu/ZnO/SiO_2_, which is deactivated after only 20 h on stream.

Apart from tuning the product selectivity and preventing active sites sintering, the core-shell catalyst can help fix and activate CO_2_ molecular activation. Hydroxyl species, which can be obtained from transition metal phyllosilicate (TM@SiO_2_p), enhance CO_2_ hydrogenation. Jangam et al. [94] prepared Cu-SiO_2_p via a hydrothermal method and compared its performance with conventionally impregnated Cu-SiO_2_ for CO_2_ hydrogenation to MeOH at 200–350 °C. Cu-SiO_2_p reduced at 225 °C produces a stable CO_2_ conversion and methanol selectivity of 3.5% and 77%, respectively.

Recently, hollow Cu@ZrO_2_ derived from a MOF network has been developed through pyrolysis for selective CO_2_ hydrogenation to methanol [101]. The hollow structure provides easy access of CO_2_ and H_2_ to diffuse on active sites. Han et al. found that the basic sites of Cu-ZrO_2_ interfaces are responsible for the main adsorption and activation sites of CO_2_. The core-shell confinement structure yields a high methanol selectivity of 85% at 220 °C.

Other than Cu-based catalysts, noble metals, such as Pd-based heterogeneous catalysts also received recognition for CO_2_ hydrogenation. The electronic structure of Pd-based catalysts plays significant roles in the reaction because its metallic sites can be tuned to obtain high-stability catalysts. However, their applications for large-scale plants are limited by sintering and expensive source. Xiao et al. [102] have recently designed stale Pd NPs in a confined environment. Pd@Cu core-shell was confined within a layered double hydroxide through modified coprecipitation. They conducted a catalytic test on formate species formation to identify the feasible CO_2_ hydrogenation pathway without forming CO intermediates. Pd_0.4_@CuMgAlO_x_ with a CO_2_/H_2_ composition of 20:20 successfully yielded 5.68 mmol∙g1∙h-1 of formate, whereas the core-shell catalyst showed no significant loss in formate yield after the fourth cycle, confirming its excellent stability. Kinetically, the Pd metallic sites govern the H_2_ dissociation by forming active Pd-H.

Table 2 summarizes recent investigation on application of core-shell nanostructured catalysts in CO_2_ hydrogenation to methanol made by research groups during the last five years. Most of the studies on CSN application were found to exhibit better catalytic performance compared to the traditional catalysts preparation method. The overall hydrogenation of CO_2_ conversion reaction enhanced the stability and activity of metal-based catalyst, which acts as the core-active metal. The confinement effect of metal catalyst restrains fast deactivation issues such as copper sintering, which contributes toward pore blocking from size-selective reactant chemisorption, thus allowing more conversion of CO_2_ and H_2_ dissociation in CSN catalysts, leading to greater production of the selective product, methanol.

### 3.2. CO_2_-Reforming Reactions

Although renewable H_2_ production via electrolytic, photoelectrochemical (PEC), and solar thermochemical methods is promising, cost effective, abundant, and sustainable, it is still not viable for industrial commercialization. Thus, thermocatalytic hydrogen production, which is a nonrenewable route that is effective for rich H_2_ production from biomass, is still relevant in the gas production market. The reforming of fossil fuels, especially natural gasses, via decomposition of hydrocarbon molecules to release H_2_ is the most common source for H_2_ production globally. Traditionally, H_2_ can be produced via several processes, such as steam methane reforming, partial oxidation reforming, methane pyrolysis, coal gasification, and DRM.

#### 3.2.1. CO_2_ Dry Reforming of Methane

Unlike the abovementioned methods, DRM offers low operating cost, utilizes two hazard greenhouse gases (CO_2_ and CH_4_) to produce highly pure gas (CO/H_2_), and allows easy processing of value-added hydrocarbons and chemicals via the Fischer–Tropsch process [104]. DRM is a more economical process relative to other methods because it eases the gas separation of final products. CO_2_ utilization has a significant influence on DRM performance, considering that the adsorption isotherm and its activation are the main steps to achieve optimal H_2_ production.

(6) $${\mathrm{CH}}_{4}+{\;\mathrm{CO}}_{2}=2\mathrm{CO}+2H_{2};\Delta H_{298K}=247\;\mathrm{kJ}/\mathrm{mol}$$

(7) $${\mathrm{CH}}_{4}\;↔C+2H_{2};\;\Delta H_{298K}=74.9\;\mathrm{kJ}/\mathrm{mol}$$

DRM is an endothermic reaction that requires excessive heating (>700 °C) driven by the following main reaction (Equation (6)). Poisoning, catalyst deactivation, and coke deposition are the common issues faced in DRM at high temperature because of methane decomposition (Equation (7)), Boudouard reaction (Equation (5)), and CO_2_ hydrogenation (Equation (1)), whereas the RGWS reaction usually occurs in DRM at temperatures below 800 °C. To limit the RWGS reaction, DRM must operate at high temperatures, approximately 900 °C, to achieve high yields of H_2_ and CO. Hence, key factors of efficient and feasible DRM reaction are optimized temperature, pressure, CH_4_/CO_2_ ratio, and catalyst design and composition.

An effective DRM reaction mechanism theoretically involves multidisciplinary transitional states, such as methane dissociative adsorption, CO_2_ dissociative adsorption, hydroxyl group formation, and intermediate oxidation and desorption [105]. In detail, CH_4_ gases must dissociate on the catalyst surface sites to complete their tetravalency, whereby CH_3_ molecules are adsorbed on top of active metal atoms while another CH_2_ molecule occurs between two metal atoms, called step sites. Then, another greenhouse gas, CO_2_, breaks its double bond, where C-O is adsorbed on the surface between the metal and support, leaving one oxygen atom exposed. Later, the H_2_ molecules migrate from the metal particles to the support atoms to form hydroxyl (^−^OH) species at temperatures below 800 °C. Finally, the metal-surface oxygen provided by high oxygen mobility support reacts with the S-CH_x_ species to form new S-CH_x_O intermediates, which potentially form as CO and H_2_. This kinetic reaction of DRM is influenced by surface electronic properties.

Efforts have been exerted to develop novel catalysts that can increase CO_2_ and CH_4_ activities at low temperatures. Nickel is the most prevalent metal-based catalyst because of its abundancy and low cost, making it suitable for industrial catalytic processes; however, Ni is rapidly deactivated because of high carbon formation from side reactions, either methane cracking or CO disproportionation [105,106] Noble metals, such as Pd [106], Ru, and Pt [107,108,109,110], are suggested to replace Ni because they are highly resistant to carbon formation. However, their expensiveness restricts their promising properties in the larger market. Hence, catalysts that minimize coke formation and preserve the active sites in DRM need to be developed.

Core-shell catalysts may offer high thermal stability, sintering resistance, and several functionalities, which aid in reducing the rate of carbon deposition [111,112]. The core-shell structure also promotes good control of dispersion and preservation from metal agglomeration, resulting in enhanced catalyst stability. Porous materials with high thermal resistance and optimum porous size channels that could anchor the active metal core are ideal to stabilize the metal NPs and minimize metal sintering at elevated temperatures [113].

##### Core-Shell Nanostructured Catalyst for CO_2_ Reforming of Methane

Several studies on CSN catalysts for DRM have been published. Metals confined with mesoporous channels are the most extensively investigated catalysts in this field. Metal–support interaction is important in driving high activity of DRM; thus, a strong interface relationship is often demanded to inhibit active metal agglomeration [114].

Ni NPs embedded into zeolitic materials, such as silicalite, have been widely accepted as a promising structure for Ni confinement, but over 20 wt% Ni loading decreases the dispersal and encapsulation. Recently, Liu et al. have developed a controlled “dissolution–fractional crystallization” method of confining high loading and uniform Ni NPs into the hollow silicalite-1 (S-1) shell [115]. Active species of Ni as core has maintained its particle size to ca. 4–5 nm with optimized Ni loading from 3% to 20%, and the TOF remains at ca. 60 s^−1^ (800 °C) in the dry reforming of methane reaction. The augmented density of active sites with Ni loading renders an outstanding reaction rate of 20.0 mol_CH4_/g_cat_/h over 20% Ni@S-1.

A multiple core-shell structure using indium–nickel (In-Ni) intermetallic alloy as core and SiO_2_ as porous shell has been successfully synthesized using the Stober method [116]. The InNi@SiO_2_ CSN displays superior coking resistance for DRM reaction by minimizing the amount of carbon formation in DRM reaction. Even for the sample with only 0.5 wt% In doping, In_0.5_Ni@SiO_2_ CSN has been achieved based on the balance of coke deposition resistance and DRM reactivity. Hypothetically, lowering in loading confers coke resistance, whereas high In loading leads to low catalytic activity because of the formation of InNi_3_C_0.5_ species. Moreover, the specific surface area of the core-shell catalysts does not change in structural behavior even after reduction during the reaction for 20 h, indicating that no observed coke blocks the pore channels during the long-term thermal operation. The increase in electron-cloud density on Ni can weaken the ability of Ni to activate the C–H bond and decrease the deep cracking of methane. The binding energy of Ni_2p_^3/2^ in the InxNi@SiO_2_ catalyst decreases with increasing in loading, which means the interaction between Ni and Ni is weakened, and the interaction between Ni and In is enhanced, indicating that Ni particles are not easy to be sintered.

Lu et al. [117] have developed a novel structure catalyst of Ni@S2-T with Ni NPs highly dispersed in silicalite-2 zeolite (S2) via a two-step method involving the microemulsion method followed by solvent-free crystallization. S2 is a silica analog of aluminosilicate zeolite (ZSM-11), which consists of four- to six-member rings chained to create a porous channel system with 10-member ring openings. The unique pore structure and channel of S2 lattice can improve the confinement efficiency by strengthening the metal–support interaction caused by the formation of Ni phyllosilicate intermediate in the shell, which is regarded as the main reason for the superb catalytic performance of Ni@S2-T for DRM. Compared with Ni-SiO_2_ prepared by microemulsion, Ni/S2 by impregnation, and Ni@S2-O by direct crystallization, Ni@S2-T catalyst exhibits optimal catalytic activity and stability for DRM. The catalyst achieves long-term stability, over 100 h, as the conversion of CH_4_ maintains its value of 25%. Furthermore, after the Ni@S2-T catalyst is overspent, hardly any coke can be found after the prolonged test, which indicates the remarkable anti-coking ability of Ni@S2-T.

Kong et al. controlled the porous size of SiO_2_ channel under intrinsic hydrothermal treatment to allow precise control over the Ni surface [118]. The synthesized Ni@SiO_2_ CSNs show that the confinement structure can destroy large Ni ensembles and form metastable Ni−O⋅Si centers. CH_4_ activates on the small fraction of Ni to form CHx rather than carbon. Moreover, the Ni−O⋅Si center stabilized by interfacial confinement provides labile oxygen to oxidize CH_x_. This Ni catalyst exhibits highly stable activity under 800 °C, CH_4_: CO_2_ = 2:1, and 5 bars without carbon deposition for 100 h, where carbon formation is thermodynamically much favorable.

Another breakthrough of core-shell structures defined as hollow nanostructures has been intensely researched for their delimited cavity and enclosed shell [119], which could manifest tunable focal properties aside from well-defined active sites, thus enhancing the catalytic functionality. Herein, Kosari et al. modified Ni-SiO_2_ hollow spheres (HSs) with different shell thicknesses and interior cavity sizes via a hydrothermal method [120]. As a result, final hollow Ni-SiO_2_ exhibits varied shell thicknesses from 11 nm up to double-shell morphology with 77 nm being the inner shell distance. NiHS-SiO_2_-S renders CH_4_ conversion equal to 69%, which is higher than those of other low Ni-loaded catalysts. However, DRM activity rate decreases as the shell thickness of SiO_2_-derived samples is increased further. Interestingly, no coke formation during the reactivity tests indicates that the formulated NiHS-SiO_2_ catalyst is a promising candidate to catalyze the DRM reaction while acting as a strong carbon resistance material.

Recently, Marinho et al. have developed a core-shell confined structure, a Ni-based mesoporous mixed CeO_2_-Al_2_O_3_ oxide catalyst by top–down synthesis, and an evaporation-induced self-assembly (EISA) method to overcome Ni particle sintering in high-temperature DRM reaction [80]. EISA utilizes mesoporous bimetallic oxides to control the shape and robustness of the Ni@CeO_2_/Al_2_O_3_ catalyst by presenting as mesoporous structures with highly dispersed Ni in the form of NiAl_2_O_4_ spinel clusters. Small (<5 nm) and homogeneous metallic Ni particles form after reduction steps. In addition, the Ce species in the structure reinforce the strong metal–support interaction with Al_2_O_3_, which enhances oxygen mobility and acts as spatial sites for CO_2_ adsorption, thereby increasing the catalytic activity and promoting the carbon removal mechanism. Therefore, Ni@CeO_2_-Al_2_O_3_ catalysts prepared by one-pot EISA exhibit high activity and stability for DRM owing to the successful encapsulation of Ni particles and coke resistance.

Wang et al. [121] prepared basic metal oxide (MgO and La_2_O_3_)-modified Ni confined in dendritic mesoporous silica catalysts (Ni-MgO@DMS and Ni-La_2_O_3_@DMS) via a sol–gel method. The Ni-MgO@DMS CSN formed exhibits a completely confined structure and yields optimum conversion rates of CH_4_ and CO_2_ up to 35% and 40%, respectively, in a low-temperature DRM reaction of 550 °C. Remarkably, only a few insignificant carbon depositions are observed during the 8 h time-on-stream stability evaluation, which can be attributed to the fast alkaline oxides adsorption and CO_2_ activation. Moreover, the modified Ni-MgO@DMS and Ni-La_2_O_3_@DMS CSNs show high Ni sintering and carbon resistance owing to the high oxygen vacancy facilitated by the presence of magnetic oxide species Mg and La, contributing to its hydrocarbon species (CHx) activation. As the reaction time is extended to 50 h, the spent Ni-La_2_O_3_@DMS catalyst has a negligible amount of carbon formation.

Park et al. synthesized size-controlled NPs with a successful Ni NP size distribution of 5 nm anchored within SBA-15 channels [122]. The double-step hydrothermal synthesis of Ni@SBA-15 CSN was prepared using deionized water, P123, and acetic anhydride as controlling agents, followed by thermal treatment in an autoclave reactor over 35 °C for a day. The Ni@SBA-15 CSN catalyst formed in the DRM reaction shows three significant contributions toward the catalytic performance: effectively dispersing the organic-stabilized nickel NPs, preserving Ni NPs from fast sintering due to thermal aggregation, and inhibiting carbon deposition on the catalyst, thus enhancing the overall catalytic stability over 100 h conversion with corresponding CH_4_ and CO_2_ conversion rates of 73.5% and 83.1%, respectively. Notably, the optimal Ni(10)@SBA-15 with 10 wt% Ni loading spatially confined within the SBA-15 inner surface is selectively and homogeneously distributed with minor depositions on the outer SBA-15 surface. The spatial confinement effects are realized due to the strong core-shell interfaces between Ni NPs with SBA-15 surfaces that can resist the migration of active species and carbon deposition, hence generating long-term stability of DRM at 800 °C over 120 h. Table 3 is a summary of research work noted for the CSN catalyst in the DRM reaction, published during the period 2018 to 2022.

## 4. Challenges and Outlook

In catalytic reactions, preventing the rapid deactivation of catalysts is crucial to ensure high overall performance of the reaction, catalytic activities, and product selectivity. Catalyst deactivation mechanisms include sintering, poisoning, and insufficient defect site formation, which often become the major factors that inhibit large-scale applications of catalysts because of their high costs. Given that CSNs have unique morphology, tunable structure, and material storage capacity, the study of CSNs in tribology will eventually bring great breakthroughs in the future. As reported, the synthesis of conventional supported catalysts results in broad heterogeneities in their structures (agglomeration, less uniform dispersion of particles, weak metal-support interaction), but core-shell synthesis provides a high degree of uniformity of the arrangement of the catalytic functions, thus allowing great control of catalyst performance and selectivity.

However, to effectively apply such core-shell catalysts for CO_2_ thermocatalytic conversions, several points in terms of feasibility, complexity, and kinetic dynamics must be considered. The preparation methods of these advanced CSN catalysts remain challenging. Further in-depth experimental works are warranted to optimize confinement techniques for inhibiting the aggregation of core active NPs. Suggested CSN synthesis methods that have been previously reported include double-step template-assisted synthesis, one-pot synthesis, addition of capping agent step, and epitaxial growth synthesis. These different methods have inherent desirable benefits, but some significant challenges remain. For instance, multiple-step synthesis and addition of PVP as a surfactant might suppress its practical application, which is costly for long-term synthesis operation. CSN is potentially a rip-off compared with current commercialized conventional catalysts. Viability and economic challenges remain to be addressed for this area to increase the application scale of these catalysts. In addition, epitaxial growth synthesis needs an additional layer preparation to form sandwich-like CSN. Hence, one-pot synthesis might be the promising and affordable method to form CSNs, but the lack of functionality control and encapsulation make CSNs less attractive for upscaling. Solvothermal or hydrothermal procedures have been implemented as facile methods to synthesize controlled size and shape of core-shell catalysts. Despite its lower requirement for chemicals involved in the procedure, this method must operate at high temperatures and more than 10 h to ensure that the nucleation growth and dispersity of NPs are controlled in specified shapes. Moreover, encapsulation by metal oxides such as ceria, alumina, and silica require large volumes of reagents and robust catalysts to sustain shear forces in handling industrial reactors to scale-up with attractive economics.

CSN catalysts enhance the catalytic performance of CO_2_ hydrogenation to methane and CO_2_ DRM reactions. The encapsulation of active NPs within high shell porosity causes high reactant diffusion, thereby resulting in high selectivity of methane yields (~100%). Core-shell catalysts also show high stability in long stream operations and form less coke deposition. Despite its inherent benefits, the CO_2_ rate of conversion remains low as the reactant molecules deactivate rapidly in the reaction.

Although several core-shell catalysts have been developed for CO_2_ methanation and DRM, reports on such catalysts for CO_2_ hydrogenation to methanol reaction are lacking. This lack of reports can be primarily ascribed to the insufficient fundamental understanding of enhanced CO_2_-to-methanol activity and mechanism. CSN catalysts for methanol synthesis perform significantly better than conventional supported catalysts, but the metal active elements or other metals that participate in reaction catalysis remain unclear. Furthermore, the interactions between Cu core and various support shells as well as reactants with respect to their roles in CO_2_ hydrogenation to methanol are still not fully understood.

The applications of CSNs in heterogeneous catalysis are still limited by difficulties in controlling the size of shell pore channels, the size of internal cavities, and the dispersion of active metal NPs. The homogeneity of CSN components is hard to regulate. The developed CSN bottom-up fabrication (solvothermal, hydrothermal, and Stober sol-gel) involves tedious preparation steps, such as addition and removal of templates, which lead to high-cost synthesis, long synthetic duration, and difficulty for scaling-up. The one-pot method is only applicable to the preparation of materials but lacks versatility of material production. Moreover, the one-pot method often emphasizes the significance of material addition sequence. Meanwhile, top-down fabrication (ball milling, arch sputtering, etching) ignores numerous minor issues but mainly suffers from difficult control of the morphology and spatial distribution of NPs and complete NP encapsulation into voids, although great efforts have been exerted to improve these issues. Therefore, a facile and efficient method to construct core-shell catalysts still need to be developed.

## 5. Conclusions

The type of catalysts is crucial to catalytic reactions. Current heterogeneous tandem catalysts are divided into two categories: conventional heterogeneous catalysts and core-shell nano/microstructured catalysts. We summarized and illustrated the thermocatalytic behavior and functionalities of CSN catalysts and their applications in CO_2_ TC utilization. These significant achievements indicate that core-shell catalysts can be used as novel and efficient photocatalysts for CO_2_ hydrogenation (methanation, methanol synthesis, and Fischer–Tropsch) and CO_2_ dry reforming reactions.

Therefore, a facile and efficient mass production process suitable for industrial scale needs to be developed. Despite great achievements in the preparation of core-shell catalysts, challenges remain in the development of core-shell structures for hydrogenation reactions. These challenges can be summarized into three main aspects:

Considering three prior challenges and the literature we reviewed in this paper, we list the possible applications of CSNs in CO_2_ thermocatalytic conversions as following:

First, CSN catalysts are suggested to be engineered intrinsically according to specific needs or targets of heterogeneous catalytic reactions. For example, in methanol synthesis, Cu-based core-shells with anti-sintering properties can be proposed for high and low temperatures, which could guide the development of industrial methanol synthesis catalysts. Optimum active Cu-metal loading anchored inside the porous shell helps lower the preparation cost by removing the use of promoters or additives. Thickness of shell layers can be controlled by tuning the composition of the core-shell, which provides sufficient regulation for size and structural morphology of CSNs. Some transitional metal-based catalysts require special requirements to avoid rapid deactivation. Hence, preparation parameters of catalytic structures with deactivation resistance functionalities, starting from the selection of fabrication method to electronic property alteration and reaction condition parameters, need to be controlled. In direct CO_2_ hydrogenation reactions, product selectivity is always a priority because of wide product distribution in different hydrogenation pathways. Efforts should be devoted to creating reactant size selective CSNs with the specific porous shell medium. The pore channels of shells should be tailored only up to reactant molecule sizes by controlling the synthesis conditions, including calcination temperatures, application of acidic or base-assisted agents, and aging step. Ultimately, stabilization of active metal species is important to enhance catalytic performance.

Instead of experimental investigations, mechanistic studies must also be conducted to optimize nanoreactors using CSNs. Many advanced theoretical models can be applied to observe the specific active sites, surface electronic density, selective reaction pathways, and microkinetic calculations. For example, a Cu@Pd catalyst was designed to investigate the mechanism of interfacial effect in CO_2_ hydrogenation to methanol with the help of DFT calculations and experimental results. After becoming familiar with the reaction mechanism, these studies would be beneficial toward the rational design of high-performance nanoreactors and their applications to thermocatalytic CO_2_ hydrogenation and dry reforming reactions.

## Figures and Tables

**Table 1:** Catalytic performance of core-shell catalysts in CO_2_ hydrogenation to methane.

Catalysts	Fabrication Method	Reaction Conditions		SA_BET_ (m^2^ g^−1^)	Catalytic Performance			Ref.
		Pressure (Bar)	Temperature (°C)		X_CO2_ (%) ^a^	S_CH4,_ (%)	STY_CH4_ Yields (μmol_CH4_⋅ g_cat_^−1^⋅s^1^)	
Ni_7_Fe@C	Pyrolysis Wet impregnation	1	350	112.68	72.3	99.3	-	[77]
Co/MnO@PGC	Pyrolysis	1	240	163.50	32.1	99.6	13.34	[78]
Ni/Al@Al_2_O_3_	Hydrothermal surface oxidation (HTSO)	1	500	172.00	-	-	7.21	[82]
Co@mSiO_2_ Co@Silicalite-1	Solvothermal	1	400	-	56.3 43.4	80.4 48.2	48.5 20.3	[60]
Ni@Silicalite-1	Selective desilication via solvothermal	1	450	367	38.3	60	-	[83]
Ni-p@CeO_2_ (NPS-180-5C)	Hydrothermal	1	450	18.2	75.0	92.0	-	[81]
Ni/Al@MnAl_2_O_4_ Ni/Al@MgAl_2_O_4_	Deposition–precipitation	1	300	129 171	90 90	99 99	- -	
Ni@MCM-41 Ni/0.05Mg@MCM-41 Ni/0.1Mg@MCM-41	In situ hydrothermal	10	350	622.5 606.3 498.5	89 84.3 80	90 95 97.8	-	[87]
Ni@SiO_2_	Stober	15	450	263	-	89.8	-	[88]
Ni@mpCeO_2_	Solvothermal	-	350	131	80	99	-	

^a^ X_CO2:_ CO_2_ conversion, S_CH4_: methane selectivity, STY_CH4_: space-time yield.

**Table 2:** Catalytic performance of core-shell catalysts in CO_2_ hydrogenation to methanol.

Catalysts	Fabrication Method	Reaction Conditions		SA_BET_ (m^2^⋅g^−1^)	Catalytic Performance			Ref.
		Pressure (Bar)	Temp. (°C)		X_CO2_ (%) ^a^	S_MeOH,_ (%)	STY_MeOH_ (g_MeOH_⋅g_cat_^−1^⋅h^−1^)	
Cu/ZnO@UiO-bpy	-	40	250	117.8	3.3	100	2.59	[96]
Cu@ZnO_x_ Cu@ZnO_x_/ZnO	Coprecipitation	30	250	- -	3 -	100 100	4.6 146.0	[99]
Cu@SiO_2_ In@SiO_2_ CuIn@SiO_2_	Double-step solvothermal	1	250	204.2 206.6 161.6	6.5 4.3 12.5	54.2 89.0 78.2	2.40 2.56 6.55	[57]
Cu@mSiO_2_ CuZnO@mSiO_2_	One pot Solvothermal	50	250	618 589	10.2 9.8	26.5 66.6	56.6 136.6	[100]
Cu-SiO_2_ phyllosilicate	Solvothermal	1	225	159.7	3.5	77	-	[94]
Hollow Cu@ZrO_2_	Hydrothermal	30	220	614.5	5	85	144	[101]
Pd_0.4_@CuMgAlO_X_	Stober	40	100	263	-	89.8	-	[102]
Cu-ZnO@MVmSiO_2_	Stober	30	240 280		- -		23.0 34.0	[103]
Cu-ZnO-Al_2_O_3_@MVmSiO_2_		30	240 280		- -		14.2 21.6	
Cu-ZnO-ZrO_2_@MVmSiO_2_		30	240 280		- -		34.2 72.0	

^a^ X_CO2_: CO_2_ conversion, S_MeOH_: methanol selectivity, STY_MeOH_: space-time yield.

**Table 3:** Summary of catalytic performance of core-shell in DRM.

Catalysts	Fabrication Method	Reaction Conditions		Catalytic Performance					Ref.
		Pressure (Bar)	Temp. (°C)	X_CO2_ (%)	Y_CH4_ (%)	Products H_2_/CO	Reaction Rates (mol_CH2_/g_cat_/h)	Coke Form. (wt%)	
20% Ni@S-1	Dissolution-recrystallization	1	800		81.2	<1.0	20.0	3.3	[115]
In_0.5_Ni@SiO_2_	One pot microemulsion	1	800 550	98 34	93 18	1.1 -	- -	- -	[116]
5% Ni@S2-T	Hydrothermal	1	700 800	75 -	75 95	0.99 -	40.2 -	1.1 -	[117]
Ni@S-1	Hydrothermal	1	800	-	71	-	-	0	[118]
Ni-HSs/SiO_2_	Hydrothermal	1	750	80	69	0.77	-	30	[120]
10Ni@CeO_2_/Al_2_O_3_	EISA	5	800	82	71	0.88	-	0.0	[80]
Ni@Al_2_O_3_									
Ni/MgO@DMS	Sol–gel	1	800 550	96 38	88 35	0.99 0.69	- -	0 -	[121]
Ni/La_2_O_3_@DMS	So–gel	1	550	40.5	35	0.71	-	1.13	[122]
Ni(10)@SBA15	Solvothermal	1	800	83.1	73.5	0.87	-	0.9	[122]
